# Efficient Synthesis of 2-Amino-6-Arylbenzothiazoles via Pd(0) Suzuki Cross Coupling Reactions: Potent Urease Enzyme Inhibition and Nitric Oxide Scavenging Activities of the Products

**DOI:** 10.3390/molecules18088845

**Published:** 2013-07-25

**Authors:** Yasmeen Gull, Nasir Rasool, Mnaza Noreen, Faiz-ul-Hassan Nasim, Asma Yaqoob, Shazia Kousar, Umer Rashid, Iftikhar Hussain Bukhari, Muhammad Zubair, Md. Saiful Islam

**Affiliations:** 1 Department of Chemistry, Governament College University, Faisalabad 38000, Pakistan; E-Mails: yasmeenchem1@yahoo.com (Y.G.); mnazanoreen@yahoo.com (M.N.); shazia_hej@hotmail.com (S.K.); pdiftikhar@yahoo.com (I.H.B); zubairmkn@yahoo.com (M.Z.); 2 Department of Chemistry, the Islamia University of Bahawalpur, Bahawalpur 63000, Pakistan; E-Mail: faiznasim@hotmail.com (F.N.); asmayaqoobctn@gmail.com (A.Y.); 3 Institute of Advanced Technology, Universiti Putra Malaysia, UPM Serdang 43400, Selangor, Malaysia; 4 Department of Chemistry, Faculty of Science, Universiti Putra Malaysia, UPM Serdang 43400, Selangor, Malaysia; E-Mail: mdsaiful@science.upm.edu.my

**Keywords:** Suzuki cross coupling, benzothiazoles, urease activity, nitric oxide scavenging activity

## Abstract

In general, benzothiazole derivatives have attracted great interest due to their pharmaceutical and biological importance. New 2-amino-6-arylbenzothiazoles were synthesized in moderate to excellent yields via Suzuki cross coupling reactions using various aryl boronic acids and aryl boronic acid pinacol esters and the antiurease and nitric oxide (NO) scavenging activity of the products were also examined. The most active compound concerning urease enzyme inhibition was 6*-*phenylbenzo[d]thiazole-2-amine **3e**, with an IC_50_ value of 26.35 µg/mL. Compound **3c**, 6-(4-methoxyphenyl) benzo[d]thiazole-2-amine, exhibited the highest nitric oxide percentage scavenging at 100 µg/mL.

## 1. Introduction

Nowadays the benzothiazole moiety has great scientific importance as it is a weak base heterocyclic compound having numerous biological activities such as anti-inflammatory [1], analgesics [2], antitumor [3], antibacterial [4], antihistamines [5], suchistosomicidal [6] and antivirus properties [7]. Benzothiazoles are mostly found in medicinal and bio-organic chemistry, with multiple applications in drug discovery [8]. 2-Aminobenzothiazoles are used as reaction intermediates and reactants since the endocyclic N functions and NH_2_ are ideally situated to enable reactions with common bis-electrophilic reagents to make different types of fused heterocyclic compounds [9]. Priyanka and co-workers have recently reported the antimicrobial activity of benzothiazoles and their derivatives against Gram positive and negative bacteria. A number of 2-aminobenzothiazoles have intensely investigated as central muscle relaxant [10]. A series of *N*-bis-benzothiazole derivatives have been synthesized byKumbhare and tested for cytotoxicity studies against a mouse melanama cell line (B16-F10) and human monocyte cell line (U937,THP-1) [11]. Some 2-aminobenzothiazole derivatives have cytotoxicities towards tumor cells which are comparable to that of cisplatin [12].

Vicendo and coworkers have synthesized new class of thiol and aminothiol compounds derived from benzothiazole and thiadiazole and checked their ability to scavenge free radical (ABTS.^+^, .OH, DPPH.). Thiol derivatives with a thiadiazole structure are the most active compounds in scavenging ABTS.^+^ and DPPH free radicals. On the basis of QSAR and DFT studies of thiazole and benzothiazole molecules, it has been observed that these compounds are potential antioxidant agents and free radical scavengers [13]. Amtul *et al* reported that urease enzyme inhibition is an important area of drug research that has led to the discovery of vast variety of drugs that useful in a number of diseases. Urease inhibitors have been considered as goals for new antiulcer drugs [14]. Moreover bacterial urease has been shown to be an important toxic determinant in the pathogenesis of many clinical conditions in animals and humans. Urease is responsible for development of kidney stones and contributes to the pathogenesis of pyelonephritis, urolithiasis, hepatic and ammonia encephalopathy, urinary catheter encrustation and hepatic coma. Urea is the major reason of pathologies induced by *Helicobacter pylori* (HP), which permits HP to survive during colonization at the low pH of the stomach [15].

The Suzuki cross coupling reaction has developed as a powerful method in organic synthesis [16]. The main advantage of choosing Suzuki cross coupling reactions as compared to other coupling methods [17] is the availability of different aryl boronic acids that are environmentally safer and the milder reaction conditions than those needed for other organometallic reagents [18]. For the synthesis of biaryl systems the palladium-catalysed Suzuki cross coupling reaction represents one of the best methods [19]. Piscitelli and coworker reported the treatment of 4-substituated anilines with potassium thiocyanate in the presence of bromine in acetic acid for the synthesis of 6-substituted 2-amino-benzothiazoles and further their Suzuki cross coupling reactions in the presence of Pd(PPh_3_)_4_ under microwave conditions to yield phenyl-substituted 2-aminobenzothiazoles [20]. After reviewing the literature it has been found that oxadiazoles/thiadiazoles and triazoles class of compounds are the novel competitive inhibitors of urease enzyme [21]. Similarly thiazole compounds were found to present moderate to potent activity in a NO scavenging protocol [22]. The main focus of the present research work is directed to the novel synthesis of 2-amino-6-arylbenzothiazoles by utilizing the basic Suzuki cross coupling chemistry and to investigate the antiurease, NO activity of the products on the basis of previously studied hetroaryl molecules as strong urease inhibitors and NO scavengers. 

## 2. Results and Discussion

### 2.1. Chemistry

**Scheme 1:**
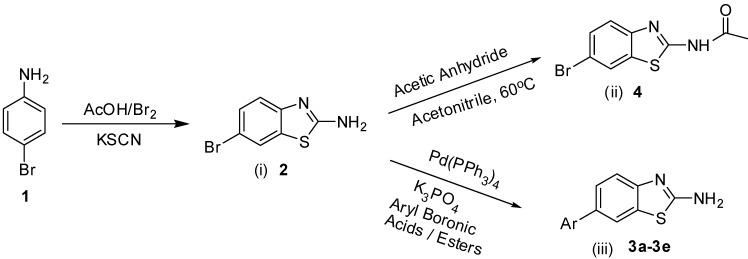
Synthesis of Benzothiazoles **2**, **4**, **3a**–**e**.

Majo and coworkers have been reported Suzuki cross coupling reactions of 2-bromobenzothiazole with aryl boronic acids to provide the corresponding 2-arylbenzothiazole compounds [23]. We have investigated the Suzuki cross coupling reactions of 2-amino-6-bromobenzothiazole (**2**) with various aryl boronic acids and esters under optimized heating conditions. There is a very little work on the synthesis of 2-amino-6-aryl benzothiazoles **3a**–**e** so far and limited information has been published on the urease enzyme inhibition and NO scavenging activities of 2-amino-6-arylbenzothiazole derivatives. 2-Amino-6-bromobenzothiazole (**2**) was prepared stirring by 4-bromoaniline with acetic acid (0.011 mol) and potassium thiocynate (0.044 mol) in solution of bromine (0.011 mol) in AcOH (7 mL) at room temperature (Scheme 1) [24].

(**1**) Synthesis of 2-amino-6-bromobenzothiazole (**2**), Synthesis of *N*-(6-bromobenzo[d]thiazol-2-yl)acetamide (**4**) and 2-amino-6-arylbenzothiazole (**3a**–**e**). *Reagents and conditions*: (i): **1** (0.011 mol), acetic acid (0.011 mol), potassium thiocynate (0.044 mol), bromine (0.011 mol); (ii) **2** (0.002 mol), acetic anhydride (0.0031 mol), acetonitrile (5 ml); (iii) **2** (2.183 mmol), aryl boronic acids and aryl boronic acid pinacol esters (2.401 mmol), K_3_PO_4_ (4.3 mmol), Pd(PPh_3_)_4_ (5 mol %), solvent / H_2_O (4:1), (see Table 1), 95 °C, 31 h.

Firstly, we studied the optimization of the reaction solvent. Thus, the effect of solvents on the coupling reactions between aryl boronic acids/esters and 2-amino-6-bromobenzothiazole (**2**) under thermal conditions at 90–100 °C was evaluated as shown in Scheme 1. We next focused our attention on the Suzuki cross coupling reaction of several varieties of aryl boronic acids/esters with 2-amino-6-bromobenzothiazole (**2**). Results of the Suzuki cross coupling reactions under optimized condition are summarized in Table 1.

When 2-amino-6-bromobenzothiazole (**2**) (2.183 mmol) was coupled with tolyl boronic acids (2.401 mmol) using a 4:1 toluene/water ratio in the presence of 5 mol% Pd(0) catalyst using K_3_PO_4_ (4.366 mmol) as base, product **3a** was obtained with moderate yield. However, when 1,4 dioxane was used in the the reaction solvent, 6-tolybenzo-[d]thiazole-2-amine (**3a**) was obtained after 31 h heating (Table the Suzuki cross coupling of 2-amino-6-bromobenzothiazole (**2**) with 4-methoxyphenyl boronic acid (4.366 mmol) in refluxing with DMF using 5 mol% of Pd(0) catalyst this resulted in the conversion into 6-(4methoxyphenyl)benzo[d]thiazol-2-amine (**3c**) in 64% isolated yield) (Scheme 1, Table 1). 

**Table 1:** Synthesis of 2-amino-6-arylbenzothiazoles.

Entry	Aryl Boronic Acid/Aryl Boronic Pinacol Ester	Product	Solvent/H_2_O (4:1)	Yield% [a]
1	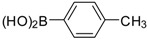	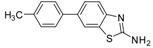 3a	Toluene	65
2	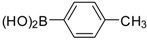	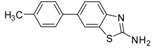 3a	Dioxane	75
3	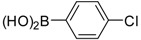	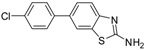 3b	Toluene	61
4	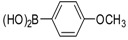	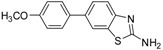 3c	DMF	64
5	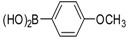	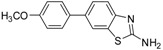 3c	Dioxane	71
6	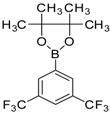	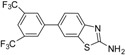 3d	Dioxane	67
7	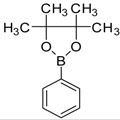	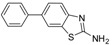 3e	Dioxane	78

[a] isolated yield Conditions: (95 °C, 31 h).

Moreover, 6-bromo-2-amino benzothiazole (**2**) was smoothly coupled with 1,3-trifluorobenzene boronic ester in 1,4-dioxane under heating conditions with conversion into the corresponding 6-(3,5 bis(triflouromethyl)phenyl)benzo[d]thiazol-2-amine (**3d**) in high yield (Table 1).

Another reaction was also carried at the same time out to obtain *N*-(6-bromobenzo[d]thiazol-2-yl) acetamide (**4**) in excellent 85% isolated yield. The reaction was carried out by the acylation of 2-amino-6-bromobenzothiazole (**2**) using a previously reported method [25] Further Suzuki cross coupling reactions of acylated 6-bromo-2-aminobenzothiazole **2** are currentlybeing studied.

### 2.2. Pharmacology

#### 2.2.1. Antiurease Activity

Hetroaryl (thiazole, triazole) pharmacophores exhibit excellent inhibitory action against bacterial as well as plant urease. Pentacyclic hetroaryl oxygen, sulphur, and nitrogen (OSN) molecules exhibit variable antiurease activity and these derivatives are planned to have these structural features: (i) a primary amine group which should compete with substrate at the acyl enzyme intermediate by means of nucleophilic substitutions during reactions (ii) a hydrophilic C- terminal to promote better diffusion into cells and to increaes affinity of inhibitor for enzyme (iii) an oxide or azole (thiazole, imidazole, pyrazole, oxazole and isoxazole) or sulfide group to enhance solubility in isotonic media [21].

**Figure 1:**
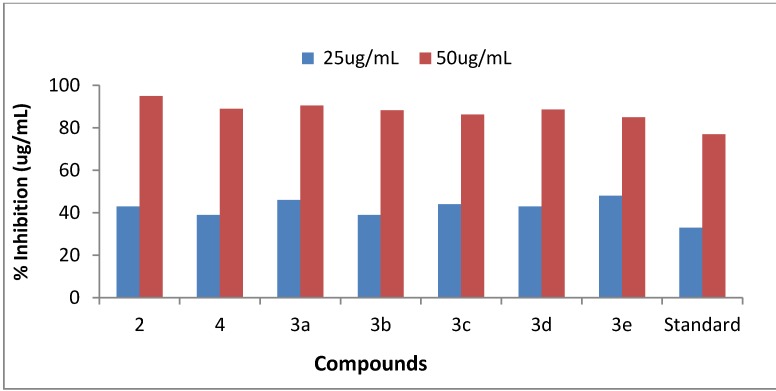
The urease inhibition activity graph.

**Figure 2:**
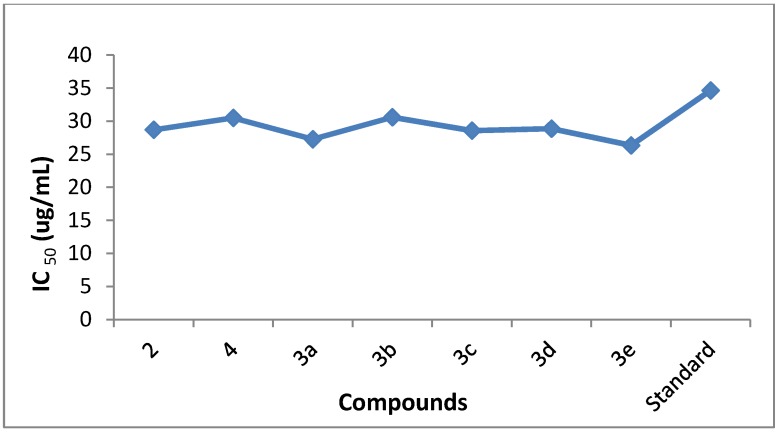
IC_50_ values of anti-urease activity.

The newly synthesized compounds **3a**–**e** were examined for their urease inhibition activities *in vitro* at 25 and 50 μg/mL concentrations. It is supposed that all these synthesized compounds have the ability to bind with the enzyme active site. Thus, enzyme stops catalyzing hydrolysis and the activity of the enzyme is inhibited. Thiourea was used as standard inhibitor, having a percentage inhibition activity of 77 ± 0.01 with an IC_50_ value of 34.65 µg/mL and the percentage urease inhibition activity was calculated according to literature protocols [26,27]. The results are presented in Table 2 (Figure 1 and Figure 2). 

**Table 2:** Urease inhibition studies of benzothiazoles.

Compound	Percentage inhibition at 25 µg/mL	Percentage inhibition at 50 µg/mL	IC_50_ (µg/mL)
**2**	43 ± 0.0034	95 ± 0.005	28.4
**4**	39 ± 0.001	89 ± 0.009	30.5
**3a**	46 ± 0.005	90.49 ± 0.002	27.27
**3b**	39 ± 0.0012	88.19 ± 0.007	30.6
**3c**	44 ± 0.005	86.24 ± 0.001	28.57
**3d**	43 ± 0.007	88.6 ± 0.002	28.88
**3e**	48 ± 0.005	85 ± 0.002	26.35
Standard	33 ± 0.04	77 ± 0.01	34.65

Values are mean ± SD of three parallel measurements.

All synthesized compounds showed very good to excellent urease inhibition activity. The compounds 2-amino-6-bromobenzothiazole (**2**) and 6-*p*-tolylbenzo[d]thiazol-2-amine (**3a**) proved to be the most potent urease inhibitors, with activities of 95 ± 0.005 and 90.49 ± 0.002 at 50 μg/mL with IC_50_ values of 28.4 and 27.27, respectively.

*N*-(6-bromobenzo[d]thiazol-2-yl)acetamide (**4**) also showed comparatively good activity, with a percentage inhibition of 39 ± 0.001 at 25 μg/mL and 89 ± 0.009 at 50 μg/mL with an IC_50_ value of 30.5. Munakata *et al.* showed that carbon chain and branch chain structure of the aryl residues apparently have no effect on activity. The additional fall in inhibitory potency may be attributed to less interaction of the surrounding residues of urease active site with the non- aromatic cyclohexane functional group [28].

Compounds **3a**,**b**,**d** exhibited significant urease inhibition activities (90.49 ± 0.002, 88.19 ± 0.007, 88.60 ± 0.002) with IC_50_ values ranging between 27.27, 30.6 and 28.88, respectively. Kobashi and coworkers reported [29] that a terminal hydroxamic acid functionality (O=C-NHOH) was evaluated against microbial and plant source urease. Fishbein and Carbone concluded that inhibitory potential of compounds was reduced with a decrease in alkyl chain length [30].

Urease inhibitions of 86.24 ± 0.001 and 85 ± 0.002 percent were seen at 50 μg/mL for 6-(4-methoxyphenyl)benzo[d]thiazol-2-amine (**3c**) and 6-phenylbenzo[d]thiazol-2-amine (**3e**). The IC_50_ values were 28.57 and 26.35, respectively. Contribution of various functional groups present on 2-amino-6-bromobenzothiazole (**2**) in urease inhibition was also investigated. In general, compounds containing electron-donating functional groups exhibited high anti-urease activity. 2-Amino-6-bromobenzothiazole (**2**) and compound **3a** containing an electron-donating methyl functional group exhibited high urease activity, whereas compound **3c** containing methoxy functional group also showed good uease anti-urease activity. These observations are in agreement with the expected results. Since urease needs Ni ions as a cofactor, compounds containing electron donating groups would be expected, though neighbouring group participation to facilitate substituent amino or imino groups to chelate these ions thereby inactivating the urease enzyme.

#### 2.2.2. Nitric Acid (NO) Scavenging Percentage (Antioxidant Activity)

Nitric acid (NO) is a free radical causing cancer [31] and inflammatory disorders [32] in the human body. As reported in previous literature, 6-flourobenzothiazole-substituated triazole compounds showed excellent antioxidant activity when measured by the DPPH method [33]. Similarly pyrozoline rings along with benzothiazole molecules exhibited very good antioxidant activity and phenyl ring present on pyrozoline enhanced the antioxidant activity against DPPH and in the ferric ion reduction method [34].

**Figure 3:**
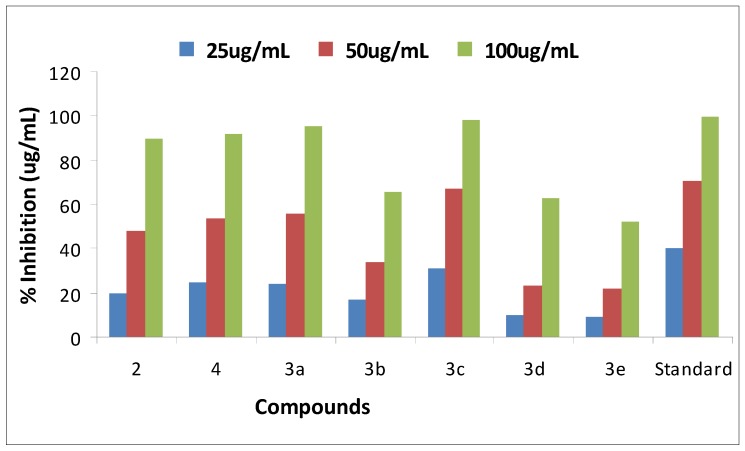
Percent nitric oxide scavenging graph.

**Figure 4:**
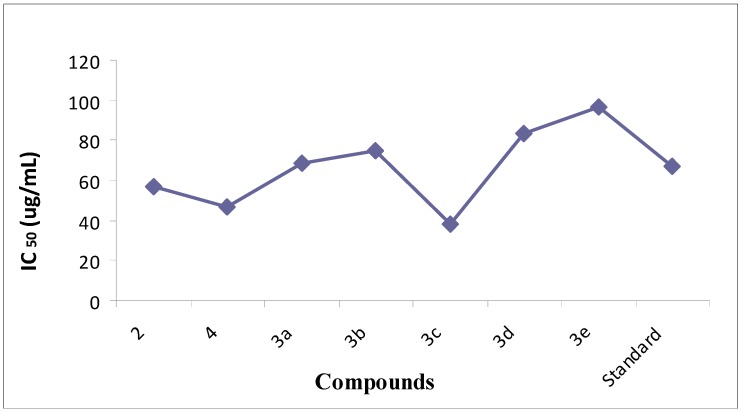
Nitric oxide scavenging activity IC_50_ values of compounds **2**,**3a**–**e**,**4**.

The newly synthesized compounds showed very good to excellent ability to scavenge NO radical [35,36]. Ascorbic acid was taken as standard with 40.5 ± 0.01 percent NO scavenging at 25 μg/mL, 70.50 ± 0.91 at 50 μg/mL and 99.5 ± 0.91 at 100 μg/mL. NO scavenging percentage activity was studied at 25, 50 and 100 μg/mL concentration, respectively. NO scavenging activities of our synthetic compounds and their corresponding IC_50_ values are shown in Table 3 (Figure 3 and Figure 4). 

**Table 3:** Antioxidant activity of synthesized compounds by nitric oxide scavenging activity.

Compound	Nitric Oxide Percentage Scavenging Activity at 25 µg/mL	Nitric Oxide Percentage Scavenging Activity at 50 µg/mL	Nitric Oxide Percentage Scavenging Activity at 100 µg/mL	IC_50_ (µg/mL)
**1** **2**	20 ± 0.004	48 ± 0.0007	90 ± 0.0007	57.8
**2** **4**	25 ± 0.001	54 ± 0.002	92 ± 0.002	46.5
**3** **3a**	24 ± 0.003	56 ± 0.001	95 ± 0.001	68.3
**4** **3b**	17 ± 0.006	34 ± 0.005	66 ± 0.005	75
**5** **3c**	31 ± 0.022	67 ± 0.002	98 ± 0.002	38.19
**6** **3d**	10 ± 0.01	23 ± 0.009	63 ± 0.009	83.75
**7** **3e**	9 ± 0.007	22 ± 0.001	52 ± 0.001	96.66
Standard	40.5 ± 0.01	70.5 ± 0.91	99.5 ± 0.91	66.66

Values are mean ± SD of three parallel measurements.

The compounds 6-(4-methoxyphenyl)benzo[d]thiazol-2-amine (**3c**) and 6-*p*-tolylbenzo[d]thiazol-2-amine (**3a**) were found to be the most potent NO scavengers with percentage activities of 67 ± 0.002 and 67 ± 0.009 at 50 μg/mL with IC_50_ values of 68.3 and 38.19 μg/mL each, respectively. *N*-(6-bromobenzo[d]thiazol-2-yl) acetamide (**4**) also exhibited comparatively good activity showing a percentage NO scavenging of 54 ± 0.002 at 50 μg/mL and 92 ± 0.002 at 100 μg/mL, with an IC_50_ of 46.5. Compounds **3a**, **3c**, **4** presented significant percentages of NO scavenging (56 ± 0.001, 67 ± 0.002, 54 ± 0.002 at 50 μg/mL) and IC_50_ values were 68.3, 38.9 and 46.5, respectively. 2-Amino-6-bromobenzothiazole (**2**) also exhibited good NO scavenging activity, 48 ± 0.0007 at 50 μg/mL and 90 ± 0.007 at 100 μg/mL with an IC_50_ of 57.8. Moderate NO scavenging percentages of 34 ± 0.005, 23 ± 0.009 and 22 ± 0.001 at 50 μg/mL were observed for 6-(4-chlorophenyl)benzo[d]thiazol-2-amine (**3b**), 6-(3,5-bis(triflouromethyl)phenyl)benzo[d]thiazol-2-amine (**3d**) and 6-phenylbenzo[d]thiazol-2-amine (**3e**) with IC_50_ values of 75, 83.75 and 96.66, respectively.

## 3. Experimental

### 3.1. General

Melting points were determined using a Buchi Melting Point B-540 apparatus. All reagents were purchased from Alfa Aesar and Sigma Aldrich company. ^1^H-NMR and C-NMR spectra was measured in CDCl_3_ and CD_3_OD on Bruker Aspect AM-400 at 400 MHz.The chemical shift was given in δ in ppm and coupling constant in *Hz*. EI-MS spectras were recorded on a JMS-HX-110 spectrometer, with a data system. For column chromatography technique, silica gel (70–230 mesh) and silica gel (230–400 mesh) were used. The reaction were monitored on TLC, using Merk Silica gel 60PF_254_ cards. The compound were visualized by UV lamp (254, 365). 

### 3.2. General Procedure for the Synthesis of 2-Amino-6-arylbenzothiazoles

For the synthesis of 2-amino-6-arylbenzothiazole **3a**–**e**, to 2-amino-6-bromobenzothiazole (**2**, 2.183 mmol), under a nitrogen atmosphere Pd(PPh_3_)_4_ (5 mol%) was added and the resulting mixture stirred for 30 min with the addition of the appropriate solvent (3 mL). After 30 min the aryl boronic acid (2.401 mmol), and K_3_PO_4_ (4.366 mmol) were added, following the addition of water (1.5 mL). The solution was stirred for 31 h at 95 °C, removed from heating and cooles to room temperature. After work up with ethyl acetate, the organic layer was separated, dried with magnesium sulfate and the solvent was removed under vacuum. The crude solid residue obtained was purified by column chromatography using a 50% ratio of ethyl-acetate and *n*-hexane to give the desired products, which were further analyzed by using different spectroscopic techniques [37]. 

### 3.3. Preparation of N-(6-bromobenzo[d]thiazol-2-yl)acetamide (**4**)

Under a nitrogen atmosphere a solution of 2-amino-6-brombromobenzothiazole (**2**, 0.00213 mol), acetonitrile (5 mL), and acetic anhydride (0.003 mol) was stirred at 60 °C for 40 min with the addition of few drops of conc. H_2_SO_4_, After 40 min distilled water (15–20 mL) was added to form a precipitate and stirring was continued for one hour at room temperature. The solution was filtered and washed with water and the product further analyzed by spectroscopic techniques [25].

### 3.4. Characterization Data

*6-Bromobenzo[d]thiazole-2-amine* (**2**). M.p. 215–217 °C; ^1^H-NMR (400 MHz, CDCl_3_ + CD_3_OD): δ = 7.80 (d, *J* = 1.8, 1H-Ar), 7.67–7.42 (m, 2H-Ar), 7.30 (s, 2H-NH_2_). ^13^C-NMR (100 MHz, CDCl_3_ +CD_3_OD): δ = 119.86, 120.35, 123.58, 124.45, 129.78, 154.0, 167.0; EIMS (*m/z* -ion mode): 228.92 [M−H]; [M-bromine and 2H fragment]^−^ = 147.00. Anal. Calcd for C7H5 N_2_S: C, 36.70; H, 2.20; N, 12.24. Found: C, 36.63; H, 2.15; N, 12.20.

*N-(6-Bromobenzo[d]thiazol-2-yl)acetamide* (**4**). M.p. 193–195 °C; ^1^H-NMR (400 MHz, CDCl_3_ + CD_3_OD): δ = 7.94 (d, *J* = 1.6, 1H-Ar), 7.61–7.53 (m, 2H-Ar), 12.20 (s, 1H-NH), 2.31 (s, 3H-CH_3_). ^13^C-NMR (100 MHz, CDCl_3_ + CD_3_OD): δ = 23.50, 117.12, 121.62, 124.17, 129.95, 146.22, 148.71, 159.20, 168.37; EIMS (*m/z* -ion mode): 271.08 [M−H^−^]; [M−Acetyl fragment]^−^ = 229.00. Anal. Calcd for C9H7Br N_2_OS: C, 39.88; H, 2.59; N, 10.34. Found: C, 39.56; H, 2.43; N, 10.29.

*6-p-Tolylbenzo[d]thiazole-2-amine* (**3a**). M.p. 184–186 °C; ^1^H-NMR (400 MHz, CDCl_3_ +CD_3_OD): δ = 8.11 (d, *J* = 7.6, 2H-Ar), 7.7–7.30 (m, 6H-Ar), 7.25 (s, 2H-NH_2_). ^13^C-NMR (100 MHz, CDCl_3_ + CD_3_OD): δ = 21.46, 116.01, 119.20, 128.57 (2C), 128.69, 132.09, 132.81 (2C), 134.0, 135.72, 140.81, 145.96, 167.85; EIMS (*m/z* -ion mode): 239.08 [M−H^−^]; [M-2H fragment]^−^ = 237.08; [M-benzene-CH_3_ fragment]^−^ = 149.17; [M−CH_3_ fragment and 2-H]^−^ = 221.25. Anal. Calcd for C14H12 N_2_S: C, 69.97; H, 5.03; N, 11.66. Found: C, 69.75; H, 4.95; N, 11.46.

*6-(4-Chlorophenyl)benzo[d]thiazole-2-amine* (**3b**). M.P, 194–196 °C; ^1^H-NMR (400 MHz, CDCl_3_ +CD_3_OD): δ = 7.59 (d, *J* = 2, 1H-Ar), 7.43–7.26 (m, 6H-Ar). ^13^C-NMR (100 MHz, CDCl_3_ + CD_3_OD): δ = 114.65, 119.27, 122.96, 123.42 (2C), 128.51, 128.91, 129.32 (2C), 132.03, 134.96, 150.02, 167.48; EIMS (*m/z* -ion mode): 259.08 [M-H^−^]; [M-NH_2_ fragment]^−^ = 243.00; [M-benzene and Cl-fragment]^−^ = 149.08 Anal. Calcd for C13H9Cl N_2_S: C, 59.88; H, 3.48; N, 10.74. Found: C, 59.78; H, 3.33; N, 10.69.

*6-(4-Methoxyphenyl)benzo[d]thiazole-2-amine* (**3c**). M.p. 165–167 °C; ^1^H-NMR (400 MHz, CDCl_3_ +CD_3_OD): δ = 7.56–7.40 (m, 3H-Ar), 7.38 (d, *J* = 3.2, 1H-Ar), 7.25 (2H-NH_2_), 3.48 (S,3H-OCH_3_). ^13^C-NMR (100 MHz, CDCl_3_ + CD_3_OD): δ = 49.57, 114.51, 118.13, 122.96, 123.37 (2C), 129.24 (2C), 131.88, 132.28, 149.38, 167.73; EIMS (*m/z* -ion mode): 255.08 [M−H^−^]; [M−OCH_3_ fragment]^−^ = 237.17; [M-CH_3_ and N fragment]^−^ = 227.08; [M-NH fragment]^−^ =242.0. Anal. Calcd for C14H12N_2_OS: C, 65.60; H, 4.72; N, 10.93. Found: C, 65.54; H, 4.57; N, 10.79. 

*6-(3,5-bis(Triflouromethyl)phenyl)benzo[d]thiazole-2-amine* (**3d**). M.p. 180–182 °C; ^1^H-NMR (400 MHz, CDCl_3_ + CD_3_OD): δ = 8.22 (s, 1H-Ar), 8.08 (s, 2H-Ar), 7.86–7.55 (m, 3H-Ar), 7.26 (s, 2H-NH_2_). ^13^C-NMR (100 MHz, CDCl_3_ +CD_3_OD): δ = 116.6, 117.5, 122.6, 123.5, 124.72 (2C), 128.5, 129.73, 132.03 (2c), 133.5, 134.50, 152.02, 168.50; EIMS (*m/z* -ion mode): 361.08 [M−H^−^]; [M-NH_2_ fragment]^−^ = 346.2; [M-2-aminobenzothiazole fragment]^−^ = 214.11. Anal. Calcd for C15H8 F_6_N_2_S: C, 49.73; H, 2.23; N, 7.73. Found: C, 99.35; H, 2.19; N, 7.69.

*6-Phenylbenzo[d]thiazole-2-amine* (**3e**). M.p. 175–177 °C; ^1^H-NMR (400 MHz, CDCl_3_ +CD_3_OD): δ = 7.59 (d, *J* = 1.6, 1H-Ar), 7.57–7.32 (m, 7H-Ar), 7.27 (s,2H-NH_2_). ^13^C-NMR (100 MHz, CDCl_3_ +CD_3_OD): δ = 115.01, 118.70, 123.57, 128.56, 128.68 (2C), 129.57 (2C), 131.91, 132.24, 132.01, 147.61, 167.85; EIMS (*m/z* -ion mode): 225.08 [M−H^−^]; [M-benzene and 2 H fragment]^−^ = 147.08; [M-benzene and NH_2_ fragment]^−^ = 135.17. Anal. Calcd for C13H10N_2_S: C, 69.43; H, 4.45; N, 12.38. Found: C, 69.39; H, 4.43; N, 12.36.

### 3.5. NO Scavenging Activity

The antioxidant activity of 2-aminobenzothiazole and it derivatives was determined by assaying Nitric oxide (NO) scavenging activity suing a test adopted from the Garrat method based on the diazotization reaction described by Griess. The assay uses sodium nitroprusside as the source of NO and sulfanilamide and *N*-1-naphthylethylenediamine dihydrochloride under acidic conditions to detect NO_2_^−^ generated at the expense of NO by the antioxidant system. Briefly, a known quantity of the test compounds **2**, **3a**–**e**, **4** in solution form was mixed with 20 mM sodium nitroprusside solution (100 µL). Total volume was made up to 1,000 µL with 200 mM Phosphate buffer, pH 7.4. The contents were mixed well and incubated at 37 °C for 2 h followed by addition of Griess reagent (100 µL). The mixture was kept at room temperature for 20 min. Optical density (OD) of the colored solution formed was measured at 528 nm. Ascorbic acid was used as the positive control while the negative control was used to develop the standard curve [36]. 

### 3.6. Antiurease Activity

The urease inhibitory activity of the synthesized compounds **2**, **3a**–**e**, **4** was determined as follows: phosphate buffer (pH 7, 200 µL) containing 1 unit of enzyme was mixed with phosphate buffer (230 µL) and of a particular stock solution (20 µL, thiourea or the test compound). The reaction mixture was incubated at 25 °C for 5 min. After the incubation period urea stock solution (400 µL, 20 mM) was added. Calibration mixture was prepared with no urea solution. All test tubes were further incubated at 40 °C for the action of urease for 10 min. This was followed by the addition of phenol hypochlorite reagent (1150 µL). Tubes were again incubated at 56 °C for 25 min. After 5 min of cooling absorbance of the blue coloured complex thus formed was noted at 625 nm and percentage inhibition was calculated. The IC_50_ values were determined using the EZ-fit kinetic data base [26,27].

## 4. Conclusions

In summary we have reported an efficient synthesis of 2-amino-6-arylbenzothiazoles and *N*-(6-bromobenzo[d]thiazol-2-yl)acetamide starting from 2-amino-6-bromobenzothiazole. A palladium catalyst was used in Suzuki coupling reactions in the presence of different boronic acids/esters. The results of this study reveal that 2-aminobenzothiazole derivatives can be used explored as anti-urease and antioxidant molecules.
